# In Reply to “Pulse of Prudence: Navigating Isolated Lung Ultrasound in Hemodialysis Patients”

**DOI:** 10.1016/j.xkme.2024.100863

**Published:** 2024-07-04

**Authors:** Emily H. Chang, Jennifer E. Flythe

**Affiliations:** 1 University of North Carolina (UNC) Kidney Center, Division of Nephrology and Hypertension, Department of Medicine, UNC School of Medicine, Chapel Hill, NC; 2 Cecil G. Sheps Center for Health Services Research, UNC, Chapel Hill, NC

We thank Bhasin-Chhabra and Koratala^1^ for their interest in our study.^2^ They underscore the limitations of lung ultrasound for differentiating between cardiogenic and pneumogenic B-lines and identifying excess fluid that is accessible to ultrafiltration with dialysis, especially in the presence of infection, right-sided heart failure, and valvular dysfunction.^3^^,^^4^ Indeed, these limitations of the interpretation of lung ultrasound led to our decision to study the utility of lung ultrasound among patients with dialysis-dependent chronic kidney failure in the hospital, a setting where infection and lung and/or cardiac pathology are common. As noted by Bhassin-Chhabra and Koratala,^1^ we attempted to limit the influence of pulmonary conditions by excluding patients with major lung pathology. However, other underlying conditions, known limitations of lung ultrasound such as operator skill and incomplete accessibility of all lung fields,^5^ as well as the imperfection of bioimpedance spectroscopy as a gold standard^6^ may have contributed to the relatively weak agreement between lung ultrasound– and bioimpedance spectroscopy–measured volume statuses in our study. We agree that additional research aimed at identifying more accurate whole-body approaches to volume assessment, including other ultrasound examinations, among hospitalized, dialysis-dependent patients is needed.
